# Provision of emergency obstetric care at secondary level in a conflict setting in a rural area of Afghanistan – is the hospital fulfilling its role?

**DOI:** 10.1186/s13031-018-0137-1

**Published:** 2018-01-22

**Authors:** Daphne Lagrou, Rony Zachariah, Karen Bissell, Catherine Van Overloop, Masood Nasim, Hamsaya Nikyar Wagma, Shafiqa Kakar, Séverine Caluwaerts, Eva De Plecker, Renzo Fricke, Rafael Van den Bergh

**Affiliations:** 1 Medical department, Mother and Child Health Unit, Brussels Operational Centre, Médecins Sans Frontières, Rue de l’Arbre Bénit 46, 1050 Brussels, Belgium; 2 Medical department (Operational Research), Operational Centre Brussels, Médecins Sans Frontières, Luxembourg City, Luxembourg; 3 International Union against Tuberculosis and Lung Disease, Paris, France; 4 Operational department, Brussels Operational Centre, Médecins Sans Frontières, Brussels, Belgium; 5 Médecins Sans Frontières, Kabul, Afghanistan; 6 Khost Public Health department, Ministry of Public Health, Khost, Afghanistan; 7 Médecins Sans Frontières, Khost, Afghanistan

**Keywords:** Maternal mortality, Deliveries, Direct obstetric complications, Newborn, MSF, CEmONC, Operational research, SORT IT

## Abstract

**Background:**

Provision of Emergency Obstetric and Neonatal Care (EmONC) reduces maternal mortality and should include three components: Basic Emergency Obstetric and Neonatal Care (BEmONC) offered at primary care level, Comprehensive EmONC (CEmONC) at secondary level and a good referral system in-between. In a conflict-affected province of Afghanistan (Khost), we assessed the performance of an Médecins Sans Frontières (MSF) run CEmONC hospital without a primary care and referral system. Performance was assessed in terms of hospital utilisation for obstetric emergencies and quality of obstetric care.

**Methods:**

A cross-sectional study using routine programme data (2013–2014).

**Results:**

Of 29,876 admissions, 99% were self-referred, 0.4% referred by traditional birth attendants and 0.3% by health facilities. Geographic origins involved clustering around the hospital vicinity and the provincial road axis. While there was a steady increase in hospital caseload, the number and proportion of women with Direct Obstetric Complications (DOC) progressively dropped from 21% to 8% over 2 years. Admissions for normal deliveries continuously increased. In-hospital maternal deaths were 0.03%, neonatal deaths 1% and DOC case-fatality rate 0.2% (all within acceptable limits).

**Conclusions:**

Despite a high and ever increasing caseload, good quality Comprehensive EmONC could be offered in a conflict-affected setting in rural Afghanistan. However, the primary emergency role of the hospital is challenged by diversion of resources to normal deliveries that should happen at primary level. Strengthening Basic EmONC facilities and establishing an efficient referral system are essential to improve access for emergency cases and increase the potential impact on maternal mortality.

## Background

An important way of reducing maternal mortality is scaling up the provision of Emergency Obstetric and Neonatal Care (EmONC) for the management of complications that arise during pregnancy and childbirth. Evidence-based strategies for providing this care promote a comprehensive approach that links primary and secondary levels of care, as follows: 1) provide accessible, acceptable and good quality Basic Emergency Obstetric and Neonatal Care (BEmONC) at the primary health care level, 2) ensure efficient referral from primary level of mothers with complications to Comprehensive Emergency Obstetric and Neonatal Care (CEmONC) provided at a secondary level facility, and 3) ensure quality of care in these CEmONC services [1–4]. This three-pronged approach has been shown to reduce maternal mortality, but little is known about the performance of the individual components when they are offered in isolation.

Afghanistan is an example of a setting where equitable access to healthcare through the simultaneous provision of these three components has not yet been possible throughout the country. The need for a comprehensive approach to improving maternal care, however, is great. Despite substantial progress in reducing maternal deaths over the past 10 years, the country continues to have one of the highest Maternal Mortality Ratios (MMRs) in the world, shown by modelled estimates to approximate 400 (range 220–750) maternal deaths per 100,000 live births [5]. Several factors are likely to be responsible for this situation, in particular the three decades of conflict that have debilitated the health and transportation infrastructure and resulted in the vast majority of women and newborns not having access to good quality care [6–8].

In 2012, with the aim of helping to reduce maternal deaths in the conflict-affected regions of Afghanistan, Médecins Sans Frontières (MSF) opened a maternity hospital in Khost province. The hospital addresses the secondary level of care components of the comprehensive approach. It focuses on providing a full package of CEmONC, but in fact provides obstetric services to all women presenting in labour, irrespective of whether they present with complications. MSF did not establish an ambulance referral system. Health Net International (HNI) is providing support to the health centres with a local non-governmental organisation (NGO), to which HNI has sub-contracted some of the work. Despite this support, the system is not yet capable of fully closing gaps in access to and quality of care [6, 9].

As little is known about how well such a CEmONC centre can function in the absence of a patient referral system and a well-functioning primary health care system, we set out to assess its performance. Specifically, we aimed to document the origin of patients, their obstetric characteristics, the proportions of emergency cases, and the quality of obstetric care including the maternal and newborn outcomes of women admitted to the maternity hospital.

## Methods

### Study design

Cross-sectional study using routine programme data.

### General setting

Afghanistan is a mountainous country in South-Central Asia with an estimated population 27 million, of whom 76% live in rural areas [10]. Literacy rates in rural areas are as low as 10% among women [11]. With 60% of the Afghan population not having access to basic health facilities in 2002 [12], the Ministry of Public Health (MoPH) established both the Essential Package of Hospital Services (EPHS) and the Basic Package of Health Services (BPHS). The EPHS and BPHS provide a framework for the provision of primary and secondary health services through different contracting mechanisms implemented with funding from main donors (USAID, European Union, World Bank) and NGOs [13]. These services are faced with several challenges, including: insecurity in some areas, shortages of female medical staff both in terms of numbers and capacity, limited or no supervision, and drug and consumable stock outs. Nationally, 60% of women deliver without skilled birth attendance and contraceptive use is low, at 13.8% [14].

Khost province in the east of Afghanistan is one of the most intense zones of conflict as it has been the heart of the Taliban insurgency for decades [15]. Population estimates vary from 546,800 [10] to one million. The inhabitants are of Pashtun ethnicity, and agriculture, trade and livestock are the main sources of revenue. In Khost, the EPHS is implemented by one provincial hospital of 100 beds – the implementation of the BPHS and EPHS is managed by HNI and its local partners. The maternity of this hospital is overstretched, with an average of 700 deliveries per month and only one midwife per 24-h shift in 2012. There are twelve comprehensive health centres but no district hospitals in the province. With the exception of one, all comprehensive health centres conduct deliveries (18 in average per month), but none of these health centres provide the full package of basic emergency obstetric care. Private clinics provide varying packages of care, and rarely obstetric surgery.

#### The MSF maternity hospital in Khost and package of emergency obstetric care

In order to improve access to comprehensive emergency obstetric care (Table 1) [16], MSF opened a 46 bed maternity hospital in 2012. An integrated but basic neonatal care unit of 14 cots is available, and family planning is offered systematically. The hospital aims to provide care mainly for women with direct obstetric complications (DOC), as defined in Table 2 – these reflect the operational MSF definitions of DOC, as diagnostics for some conditions listed as DOC in reference guides are not always available in MSF contexts (such as amniotic fluid embolism) [16]. At the end of 2014 the hospital was being run by 283 staff (272 national and 11 international), of whom 170 are medical staff, including 2 gynaecologists, 3 medical doctors (MD) with obstetric training, 2 anaesthetists, 1 MD anaesthetist, 3 nurse anaesthetists, 4 paediatricians, 72 (nurse) midwives and 7 medical assistants. In order to respect local culture, all staff are female with the exception of some specialist profiles such as anaesthetist and paediatricians. All services are provided free-of-charge.

**Table 1:** Standard CEmONC package in the MSF Khost maternity hospital, Afghanistan, 2013–2014 (expanded from [16])

For the mother
• Administration of parenteral anticonvulsants and antibiotics
• Administration of uterotonic drugs
• Manual removal of the placenta
• Balloon tamponade
• Removal of retained products following abortion (Manual Vacuum Aspiration or Dilatation & Curettage)
• Assisted vaginal delivery (vacuum extraction)
• Surgery: caesarean section, hysterectomy, laparotomy for ectopic pregnancy
• Perform blood transfusion
For the newborn
• Standard newborn care including neonatal resuscitation
• Administration of parenteral fluids, antibiotics
• Administration of oxygen

**Table 2:** Operational definitions of Major Direct Obstetric Complications in the MSF Khost maternity hospital, Afghanistan, 2013–2014 (expanded and adapted from [16])

Haemorrhage
*Antepartum*
• Severe bleeding before and during labour: placenta praevia, placental abruption
*Postpartum* (any of the following)
• Bleeding that requires treatment (e.g. provision of intravenous fluids, uterotonic drugs or blood)
• Retained placenta
• Severe bleeding from lacerations (vaginal or cervical)
• Vaginal bleeding in excess of 500 ml after childbirth
• More than one pad soaked in blood in 5 min
*Ectopic pregnancy*
• Internal bleeding from a pregnancy outside the uterus; lower abdominal pain and shock possible from internal bleeding; delayed menses or positive pregnancy test
*Ruptured uterus*
• Uterine rupture with a history of prolonged or obstructed labour when uterine contractions suddenly stopped. Painful abdomen (pain may decrease after rupture of uterus). Patient may be in shock from internal or vaginal bleeding
Prolonged or obstructed labour: (dystocia, abnormal labour) (any of the following)
• Prolonged established first stage of labour (> 12 h)
• Prolonged second stage of labour (> 1 h)
• Cephalo-pelvic disproportion, including scarred uterus
• Mal-presentation: transverse, brow or face presentation
Postpartum sepsis
• A temperature of 38 °C or higher more than 24 h after delivery (with at least two readings, as labour alone can cause some fever) and any one of the following signs and symptoms: lower abdominal pain, purulent, offensive vaginal discharge (lochia), tender uterus, uterus not well contracted, history of heavy vaginal bleeding. (Rule out malaria)
Complications of abortion (spontaneous or induced)
• Haemorrhage due to abortion which was managed medically or surgically (manual vacuum aspiration or dilatation and curettage)
• Sepsis due to abortion (including perforation and pelvic abscess)
Severe pre-eclampsia and eclampsia
• Severe pre-eclampsia: Diastolic blood pressure ≥ 110 mmHg or proteinuria ≥3 after 20 weeks’ gestation. Various signs and symptoms: headache, hyperreflexia, blurred vision, oliguria, epigastric pain, pulmonary oedema
• Eclampsia
• Convulsions; diastolic blood pressure ≥ 90 mmHg after 20 weeks’ gestation or proteinuria ≥2. Signs and symptoms of severe pre-eclampsia may be present (adapted from [16]**)**

The MSF facility operates in parallel with the provincial hospital: while it reports to the MoPH and formal memoranda of understanding have been signed with the MoPH concerning its operation, it relies on MSF staff, guidelines, and principles. No functioning referral system existed between the MoPH primary health care facilities and the MSF second level care facility.

#### Patient flow

Security constraints, cost, and distance or lack of transport have been found to be the main barriers for patients to reach the facility [7]. Most women arrive in the morning, accompanied by a male and female relative. Only women are allowed into the maternity hospital; men have to wait in a dedicated area. All women are examined by a lady health visitor (LHV, nurse assistant) and admitted if they are in labour or have a pregnancy or post-partum complication. Management is done by midwives and midwife assistants. A doctor is consulted as second-line in case of complications and performs surgery. After delivery, patients are encouraged to stay for 24 h if they have had a normal vaginal delivery, however, most stay for less then 12 h. Following a caesarean section, patients stay 3 to 4 days or more, as is clinically indicated. Healthy neonates remain with their mothers in the maternity ward to foster early breastfeeding. When sick or premature, they are admitted to the neonatal ward.

#### Quality and utilisation of emergency obstetric care (CEmONC)

Simply assessing the total number of maternal deaths out of all deliveries occurring in a hospital is not a sensitive enough marker for assessing quality of obstetric care. An alternative is to focus on all women presenting with severe obstetric morbidities, collectively termed as DOC. The number of maternal deaths related to direct obstetric complications (termed as the DOC case fatality rate) is a good indicator of the overall quality of obstetric management. Low case fatality rates suggest high quality of care, since they imply that patients with severe morbidity are unlikely to die as a consequence of their condition. Conversely, high rates suggest gaps in maternal care. Furthermore, the proportion of women with DOC treated in a facility in relation to the overall expected DOC within a pregnant population (being 15% of expected deliveries in the total population), represents the contribution of that facility to the overall need of emergency obstetric care. Analysing these indicators may help identify specific shortcomings in provision and quality of emergency obstetric care and may reveal issues related to utilisation of the service that require further investigation [16, 17].

### Study population

All women admitted to the MSF maternity hospital in Khost between January 1st 2013 and December 31st 2014.

### Data collection and statistical analysis

Data related to the study objectives were extracted from a standardized and individualized electronic register (Excel) used to monitor hospital activities; this database is used in all major maternities in MSF-Operational Centre Brussels. Data were single-entered into this register from patient files and were routinely validated by supervision staff. District level population data of Khost were obtained from the Khost Governors office and used to estimate utilisation of maternity services. The number of expected deliveries by district population was derived by multiplying the crude birth rate (39 births/1000 population) [18] by the total population of the district (*n* = 1,002,563). The expected proportion of women with DOC was set at 15% of all expected deliveries/year (approximately 5865 per year or 1466 per quarter in Khost), in line with WHO guidelines [16]. The DOC case fatality rate was calculated by dividing the maternal deaths due to DOC by the overall number of patients managed with DOCs in the MSF maternity hospital and is expressed as a percentage. A level < 1% is considered acceptable.

It is estimated that a minimum of 5% of all expected deliveries will require a caesarean section for both maternal and foetal reasons [16]. This was the basis for calculating the relative contribution of the MSF maternity hospital to caesarean coverage in Khost province. Outcomes of women and newborns were assessed at hospital exit and were standardized. The chi-square test for trend was used to assess linear trend. The level of significance was set at *P* < 0.05. The data were analysed using EpiData Analysis software (version 2.2.2.183, EpiData Association, Odense, Denmark).

## Results

### Geographical origin and utilisation of the MSF maternity hospital

**Fig. 1:**
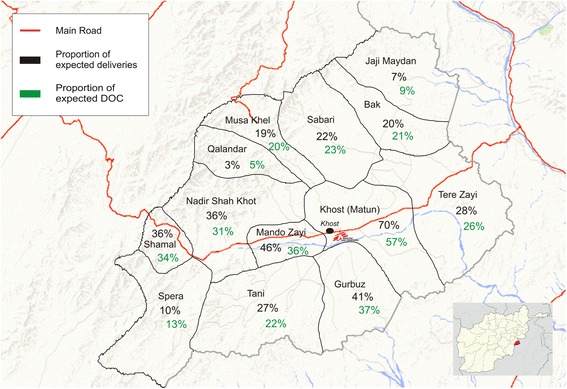
Geographical origin of women admitted to the MSF Khost maternity hospital, expressed as proportion of expected deliveries and proportion of expected DOC by district. Afghanistan, 2013–2014 (modified from Google Earth)

A total of 29,876 women were admitted to the MSF maternity hospital. Of the 29,862 women for whom geographic origin was recorded, 29,683 (99%) were from districts within Khost province, while 179 (1%) were from outside the province. Of the 29,876 admissions, 99% were self-referred, 0.4% referred by traditional birth attendants and 0.3% by health facilities. Expressed as a percentage of expected deliveries, utilisation of the MSF maternity hospital ranged from 3% to 70% between districts (Fig. 1). Women coming from districts that are clustered around the main road that passes through the province utilised the MSF hospital more than others.

### Obstetric characteristics and management of admitted women

Table 3 presents the obstetric characteristics of the 29,876 admitted women. 63% had at least one antenatal care visit during the current pregnancy. Table 4 shows the medical and surgical obstetric interventions performed: the most frequent medical interventions were Rho (D) immunoglobulin for Rhesus blood group incompatibility (given to Rhesus factor-negative women when they deliver a Rhesus factor-positive baby), instrumental vaginal deliveries, and augmentation or induction of labour. The top three most frequent surgical interventions were: episiotomy, manual vacuum aspiration for incomplete abortion, and suturing of tears. Other surgical interventions included manual removal of placenta, uterine revision, tubal ligation, and hysterectomy.

**Table 3:** Obstetric characteristics of women admitted to the MSF Khost maternity hospital, Afghanistan, 2013–2014 (*n* = 29,876)

Variable	*N*	(%)
Gravida^a^		
1	6651	(22)
2–6	16,181	(54)
> 6	6874	(23)
Not recorded	158	(< 1)
Parity^b^		
0^c^	7300	(24)
1–6	18,759	(63)
> 6	3659	(12)
Not recorded	158	(< 1)
Number of abortions		
0	24,068	(81)
1–3	5158	(17)
> 3	484	(2)
Not recorded	166	(< 1)
Attended antenatal care in the current pregnancy		
Yes	18,702	(63)
No	11,163	(37)
Not recorded	11	(< 0.5)
Rh factor		
Negative^d^	1887	(6)
Positive	27,251	(91)
Not recorded	738	(2)
Principal exit diagnosis		
Vaginal delivery without complication	23,636	(79)
Direct obstetric complications	3860	(13)
Haemorrhage	1536	(5)
Abortion with complication	1477	(5)
Prolonged and/or obstructed labour	664	(2)
Hypertensive disorders	175	(1)
Post partum sepsis	8	(< 0.5)
Preterm delivery	123	(< 0.5)
Abortion without complications	154	(1)
Other	2103	(7)

^a^The total number of pregnancies the mother has had

^b^The number of pregnancies reaching viable gestational age

^c^Parity 0 are women who never delivered, but presented with a complication in pregnancy

^d^Rh factor negative: these women required Rho (D) immunoglobulin treatment

**Table 4:** Obstetric interventions by type among women admitted to the MSF Khost maternity hospital, Afghanistan, 2013–2014

Variable	*N*	(%)
Mode of delivery		
No delivery	2528	(8)
Spontaneous vaginal delivery	25,722	(86)
Instrumental vaginal delivery	507	(2)
Caesarean section	815	(3)
Not recorded	304	(1)
Women with other obstetric interventions^a^	6390	(21)
Medical interventions		
Rho(D) immunoglobulin	1081	(4)
Augmentation of labour	364	(1)
Induction of labour	316	(1)
Obstetrical manoeuvre^b^	194	(1)
Tocolysis / pulmonary maturation	18	(< 1)
Additional medical treatment of incomplete abortion	16	(< 1)
Additional medical treatment of postpartum haemorrhage	11	(< 1)
Blood transfusion	1	(< 1)
Surgical interventions		
Episiotomy	2597	(9)
Manual vacuum aspiration	1566	(5)
Suturing of tears	1055	(4)
Manual removal of placenta	169	(1)
Uterine revision	162	(1)
Tubal ligation	74	(< 1)
Hysterectomy	68	(< 1)
Digital curettage	15	(< 1)
Balloon tamponade	7	(< 1)
Salpingectomy	4	(< 1)
Dilatation and curettage	2	(< 1)
Other laparotomy	8	(< 1)
Other intervention	21	(< 1)

^a^Excluding Caesarean section; some women underwent more than one intervention

^b^Obstetrical manoeuvres: to manage shoulder dystocia, breech delivery, OP position, etc.

### Direct obstetric complications

**Fig. 2:**
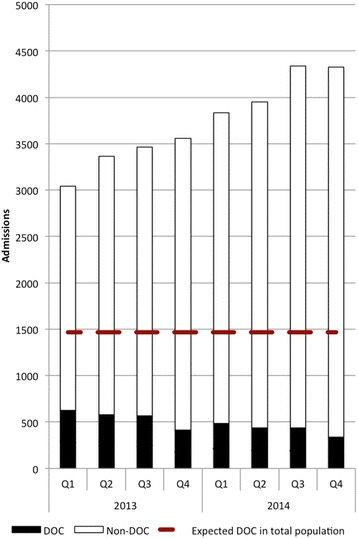
Admissions and direct obstetric complications at the MSF Khost maternity hospital per quarter, Afghanistan, 2013–2014

The total number of women with direct obstetric complications was 3890, representing 13% of all admissions over the two-year study period. Fig. 2 shows the total number of women with DOC among all admissions over time. Between Quarter 1 of 2013 and Quarter 4 of 2014, a significant decrease, from 21% to 8%, was observed (*P* < 0.001). Coverage of the overall expected DOC burden in the district by the MSF hospital was 43% in Q1 of 2013, but decreased to 23% by Q4 of 2014 (Fig. 2).

The types of DOC included haemorrhage (40%), abortion with complications (38%), prolonged and/or obstructed labour (17%), hypertensive disorders (5%), and post-partum sepsis (0.3%).

A total of 815 caesarean sections were performed, representing an estimated contribution of the MSF maternity hospital to 42% of the expected minimum 1955 caesarean sections/year in Khost province (calculated on the basis of 5% of all expected deliveries requiring a caesarean section).

### Maternal and neonatal outcomes at hospital exit

A total of 8 maternal deaths occurred in the hospital. Six of these were associated with a DOC, including post-partum haemorrhage (4), ante-partum haemorrhage (1) and severe pre-eclampsia (1), giving a DOC case fatality rate of 0.2%. The two deaths that were not associated with DOC were presumed to be: pulmonary embolism (1) and amniotic fluid embolism (1).

Table 5 shows the maternal and neonatal hospital outcomes at hospital exit. Of 29,876 women admitted, 98% were discharged. Of all births, 96% were live births. Of all live births, 3% were transferred to neonatal care and there were 1% in-hospital neonatal deaths registered in the maternity ward. Due to limitations of the existing data reporting system, it may be that not all the neonates who died in the neonatology ward were reported. Of 954 stillbirths, 84% did not have a foetal heart beat on admission.

**Table 5:** Maternal and newborn outcomes at maternity exit, MSF Khost maternity hospital, Afghanistan, 2013–2014

Variable	N	(%)
Maternal outcome (n = 29,876)		
Discharged	29,352	(98)
Referred (within and out of the facility)	71	(< 0.5)
Death	8	(< 0.5)
Absconded	435	(1)
Not recorded	10	(< 0.5)
Birth outcome (*n* = 28,130)^a^		
Live births	27,105	(96)
Discharged	25,966	(92)
Transferred to neonatal unit	749	(3)
Referred to other health facility	6	(< 0.5)
Neonatal death	184	(1)
Absconded	200	(1)
Still births	954	(3)
Stillbirth with FHR^b^ positive on admission	150	(< 1)
Stillbirth with FHR negative on admission	804	(3)
Not recorded	71	(< 0.5)

^a^Some women were admitted for pregnancy- and postpartum-associated complications, and thus not all of admitted patients delivered during their hospital stay. Additionally, some women gave birth to twins or triplets; hence, a mismatch exists between the number of maternal and birth outcomes

^b^Foetal heart rate

## Discussion

This is the first study that reports on the provision of CEmONC in a non-secure and difficult to access setting within Afghanistan. The findings show a high and steadily increasing hospital utilisation rate, despite which a high quality of care has been maintained. An issue of concern is the progressive decrease in proportions of emergency cases over 2 years compared to normal deliveries (Fig. 2). This poses a challenge to the primary role of the MSF maternity - the management of women with obstetric complications.

The sustained increase in admissions throughout the study period, resulting in high case loads, is encouraging as it suggests acceptability of the services by the general population and possibly also reflects good health seeking behaviour in the general population of Afghanistan [7]. Another very positive finding was that it is feasible to provide quality EmONC in this challenging setting, as illustrated by the acceptable maternal and neonatal outcomes, and the low overall DOC crude mortality rate - a sensitive indicator of the quality of emergency obstetric care. The maintenance of quality despite increasing caseloads could be attributed to a number of factors (Table 6).

**Table 6:** Factors possibly contributing to quality of care in the MSF Khost maternity hospital, Afghanistan, 2013–2014

• The 24-h availability of specialized maternity staff on all days of the week (including nurses/midwives, doctors, and gynaecologists).
• Infrastructure provided by MSF and adapted to provide EmONC, auxiliary services, equipment, and an uninterrupted supply of drugs and consumables.
• Ongoing “on-the-job” training by experienced midwives and gynaecologists, combined with regular refresher trainings including Advanced Life Support in Obstetric courses.
• Task shifting of specific EmONC activities from doctors and specialists (such as manual vacuum aspiration, vacuum delivery, and manual removal of placenta) to midwives, nurses, and Lady Health Visitors.
• Regular salaries paid by MSF
• Ongoing monitoring of all medical activities and analysis of outcomes to orient/define further training and human resource needs (including mortality audits, chart reviews, case discussions, etc.)

Conversely, the low and systematically decreasing proportion of DOC is a concern. It suggests that the hospital is increasingly involved with the provision of care for normal deliveries that should actually happen at the primary care level. By doing so, it is diverting hospital resources away from the target group - women with emergencies who are at higher risk of death. The malfunctioning BEmONC services at health centre level in the province and the lack of a functioning referral network are believed to be the main contributing factors. The decreasing proportion of DOC over time may also, at least partially, be explained by overburdened health staff inaccurately registering DOC conditions in patient files. This aspect needs to be verified as it may result in under-estimation or over-estimation of some DOC conditions. The same may be true for the registration of medical treatment of certain conditions, where only the medical treatments seen as exceptional (such as balloon tamponade for PPH) were systematically recorded.

There are some other interesting observations from this study. First, the considerable number of women requiring Anti-D Immunoglobulin treatment, which was the most frequent medical intervention offered. As this medication remains expensive (at 38 Euros, this hospital’s yearly budget for Anti-D Immunoglobulin is at 20,520 Euros), advocacy for price reduction and universal access would seem justified. Second, the important number of neonates in need of neonatal care highlights the vital need for providing integrated neonatal services [19–22]. Third, the very high number of stillborns may be associated with the rather low coverage of antenatal care and difficult access to the provision of skilled birth attendants during birth within an EmONC facility [23, 24]. Fourth, there was a low number of women admitted with post-partum sepsis, which is known to be one of the main direct causes of maternal mortality [25]. This may reflect poor vigilance for sepsis post discharge, and/or poor referral to the hospital for treatment.

Study strengths include the high number of individuals included in the analysis, data collected in a systematic manner, dedicated human resources for data collection from patient files and supervision.

The study has some limitations. There was a discrepancy between the population size data obtained from the Khost governorate office and from the national census. We used the former, as this provided more recently updated population figures. Another limitation was that the age of the study population was not recorded in the database. This limited the possibility of assessing outcomes of the adolescent subgroup, which is considered to be more at risk of obstetrical complications and the associated averse outcomes. Age will be included as a variable in the database henceforth.

In addition, data on maternal deaths only included hospital-based maternal mortality. Thus, there may have been some deaths that occurred after discharge, but this will require specific community level studies. It is also important to remember that this study only reports on DOCs treated in the MSF facility, not those treated in the MoPH facility. Consequently, only the MSF facility’s contribution to the EmONC met need could be calculated. Finally, the important aspect of providing family planning has not been addressed in this study. It will, however, constitute the basis of a follow-up study in the same population.

Despite the limitations, our study highlights several important observations for policy and practice. Firstly, despite the provision of high-quality CEmONC services at secondary level, the absence of a well-functioning primary care and referral system may rapidly result in overloading the CEmONC facility with normal deliveries. The CEmONC facility will then be unlikely to contribute significantly to a reduction in maternal mortality. Functioning referral systems are of the utmost importance to help the right patients reach emergency care in time. Referral systems are, however, difficult to operate when first-line structures are not optimal. Where the MoPH is unable to provide such coverage, NGOs could play an important role. Thus, the focus should be less on establishing an acceptable number of health facilities, and more on how to scale up the delivery of quality EmONC by ensuring the provision of services and medicines as well as increasing the availability of skilled staff in BEmONC signal functions [6, 7, 26].

Secondly, evidence-based interventions to reduce pregnancy and intra partum stillbirths need to be developed further [24, 27]. More effort should also be made to implement packages of evidence-based, low-tech neonatal care, including human resources, that can be offered to premature and sick newborns in settings like Afghanistan [19–22]. Finally, post-natal services have to be integrated within the system, with the aim of fostering close follow up of both mother and neonate within the first week after delivery. With hospital discharges happening early in Afghanistan for cultural and other reasons, community-based initiatives may need to be explored. This is particularly important to ensure early detection and management of sepsis.

## Conclusion

This study shows that despite a high and increasing caseload over time, it is feasible to provide good quality Comprehensive Emergency Obstetric care in a conflict setting in rural Afghanistan. Nevertheless, finding ways to create an efficient referral network with good geographic coverage as well as ensuring quality care in BEmONC and CEmONC facilities will be essential if we are to make a dent in maternal mortality in the Khost province and beyond. Unfortunately after completion of this study, another MSF hospital based in Kunduz, Afghanistan was completely destroyed by an airstrike on the 3rd of October 2015 this resulted in the death of 42 individuals including 14 MSF hospital staff [28, 29]. The 92 bed hospital which was the only facility with comprehensive trauma care capabilities for hundreds of thousands of people had to be closed. A sine qua non to upholding health services and the health system in general in such dire circumstances as Afghanistan is ensuring respect for health facilities and the protection of health workers and patients. Failure to do so would further erode confidence by patients and health personnel in improving access to, and in provision of health care.

## Abbreviations

BEmONC: Basic emergency obstetric care
BPHS: Basic package of health services
CEmONC: Comprehensive emergency obstetric care
DOC: Direct obstetric complication
EMONC: Emergency obstetric and neonatal care
EPHS: Essential package of hospital services
HNI: Health net international
MMRs: Maternal mortality ratios
MoPH: Ministry of public health
MSF: Médecins Sans Frontières
NGO: Non-governmental organisation
SORT IT: Structured operational research and Training IniTiative

## References

[1] Campbell OM, Graham WJ (2006). Strategies for reducing maternal mortality: getting on with what works. Lancet.

[2] Paxton A, Maine D, Freedman L, Fry D, Lobis S (2005). The evidence for emergency obstetric care. Int J Gynecol Obstet.

[3] Carvalho N, Salehi AS, Goldie SJ (2013). National and sub-national analysis of the health benefits and cost-effectiveness of strategies to reduce maternal mortality in Afghanistan. Health Policy Plan.

[4] Tayler-Smith K, Zachariah R, Manzi M, Van den Boogaard W, Nyandwi G, Reid T, Van den Bergh R, De Plecker E, Lambert V, Nicolai M, Goetghebuer S, Christaens B, Ndelema B, Kabangu A, Manirampa J, Harries AD (2013). Achieving the millennium development goal of reducing maternal mortality in rural Africa: an experience from Burundi. Trop Med Int Health.

[5] WHO, UNICEF, UNFPA, Bank TW, The United Nations population Division: Trends in Maternal Mortality: 1990–2013. Estimates by WHO, UNICEF, UNIFPA, The World Bank and the United Nations population Division. Geneva: World Health Organisation; 2014.

[6] Kim YM, Zainullah P, Mungia J, Tappis H, Bartlett L, Zaka N (2012). Availability and quality of emergency obstetric and neonatal care services in Afghanistan - Int J Gyn Obst 116-03 2012.Pdf. Int J Gynecol Obstet.

[7] Nic Carthaigh N, De Gryse B, Esmati AS, Nizar B, Van Overloop C, Fricke R, Bseiso J, Baker C, Decroo T, Philips M (2015). Patients struggle to access effective health care due to ongoing violence, distance, costs and health service performance in Afghanistan. Int Health.

[8] Hansen PM, Peters DH, Edward A, Gupta S, Arur A, Niayesh H, Burnham G (2008). Determinants of primary care service quality in Afghanistan. Int J Qual Health Care: J Int Soc Qual Health Care / ISQua.

[9] Nic Carthaigh N, De Gryse B, Esmati AS, Nizar B, Van Overloop C, Fricke R, Bseiso J, Baker C, Decroo T, Philips M: Patients struggle to access effective health care due to ongoing violence, distance, costs and health service performance in Afghanistan. International Health 2014 (December 2014) 7:169–175. 10.1093/inthealth/ihu086 PMC4427534 25492948

[10] AFGHANISTAN (2014). Central statistics organization ( CSO ) estimated population 2012–2013.

[11] Afghanistan National Literacy Action Plan Ministry of Education Islamic Republic of Afghanistan. 2012.

[12] UNDP (2004). Afghanistan. Millennium development goals: progress at a glance.

[13] MoPH (2005). The essential package of Hospital Services for Afghanistan. Ministry of Public Health, Kabul, Afghanistan.

[14] MoPH: Afghanistan health indicators, fact sheet. HMIS. 2014(March).

[15] Johnson TH, Mason MC (2007). Understanding the Taliban and insurgency in Afghanistan. Orbis.

[16] World Health Organization: Monitoring emergency obstetric care - a handbook. Geneva, Switzerland: World Health Organization; 2009.

[17] Ronsmans C (2009). Severe acute maternal morbidity in low-income countries. Best Pract Res Clin Obstet Gynaecol.

[18] Birth rate - Country Comparison [https://www.indexmundi.com/g/r.aspx?v=25].

[19] Zuniga I, Bergh Van Den R, Ndelema B, Bulckaert D, Manzi M, Lambert V, Zachariah R, Reid AJ, Harries AD (2013). Characteristics and mortality of neonates in an emergency obstetric and neonatal care facility, rural Burundi. Public Health Action.

[20] Darmstadt GL, Bhutta ZA, Cousens S, Adam T, Walker N, De Bernis L (2005). Evidence-based, cost-effective interventions: how many newborn babies can we save?. Lancet.

[21] Saugstad OD (2011). Reducing global neonatal mortality is possible. Neonatology.

[22] Gabrysch S, Civitelli G, Edmond KM, Mathai M, Ali M, Bhutta ZA, OMR C (2012). New signal functions to measure the ability of health facilities to provide routine and emergency newborn care. PLoS Med.

[23] Frøen JF, Cacciatore J, McClure EM, Kuti O, Jokhio AH, Islam M, Shiffman J (2011). Stillbirths: why they matter. Lancet.

[24] Bhutta ZA, Yakoob MY, Lawn JE, Rizvi A, Friberg IK, Weissman E, Buchmann E, Goldenberg RL (2011). Stillbirths: what difference can we make and at what cost?. Lancet.

[25] Say L, Chou D, Gemmill A, Tunçalp Ö, Moller A, Daniels J, Gülmezoglu AM, Temmerman M, Alkema L: Global causes of maternal death : a WHO systematic analysis. Lancet Glob Health. 2014;2(6):e323–33. 10.1016/S2214-109X(14)70227-X. Epub 2014 May. 10.1016/S2214-109X(14)70227-X 25103301

[26] Faqir M, Zainullah P, Tappis H, Mungia J, Currie S, Kim YM (2015). Availability and distribution of human resources for provision of comprehensive emergency obstetric and newborn care in Afghanistan: a cross-sectional study. Confl Heal.

[27] Pattinson R, Kerber K, Buchmann E, Friberg IK, Belizan M, Lansky S, Weissman E, Mathai M, Rudan I, Walker N, Lawn JE (2011). Stillbirths: how can health systems deliver for mothers and babies?. Lancet.

[28] Trelles M, Stewart BT, Hemat H: Averted health burden over 4 years at Médecins Sans Frontières (MSF) Trauma Centre in Kunduz, Afghanistan, prior to its closure in 2015. Surgery. 2016;160(5):1414–1421. 10.1016/j.surg.2016.05.024. Epub 2016 Jul 9. 10.1016/j.surg.2016.05.024 27407057

[29] Trelles M, Stewart BT, Kushner AL (2016). Attacks on civilians and hospitals must stop. Lancet Glob Health.

